# Range of motion and strength after surgery for brachial plexus birth palsy: 107 patients followed for 12-year

**DOI:** 10.3109/17453674.2010.539499

**Published:** 2011-02-10

**Authors:** Mikko O Kirjavainen, Yrjänä Nietosvaara, Sanna M Rautakorpi, Ville M Remes, Tiina H Pöyhiä, Ilkka J Helenius, Jari I Peltonen

**Affiliations:** ^1^Department of Orthopedsics and Traumatology and Hospital for Children and Adolescents, Helsinki University Central Hospital; ^2^Helsinki Medical Imaging Center, University of Helsinki, Helsinki; ^3^Department of Orthopedics and Traumatology, Turku University Central Hospital, Turku; ^4^Department of Orthopedics and Traumatology, Helsinki University Central Hospital, Finland

## Abstract

**Background:**

There is little information about the range of motion (ROM) and strength of the affected upper limbs of patients with permanent brachial plexus birth palsy.

**Patients and methods:**

107 patients who had brachial plexus surgery in Finland between 1971 and 1998 were investigated in this population-based, cross-sectional, 12-year follow-up study. During the follow-up, 59 patients underwent secondary procedures. ROM and isometric strength of the shoulders, elbows, wrists, and thumbs were measured. Ratios for ROM and strength between the affected and unaffected sides were calculated.

**Results:**

61 patients (57%) had no active shoulder external rotation (median 0° (-75–90)). Median active abduction was 90° (1–170). Shoulder external rotation strength of the affected side was diminished (median ratio 28% (0–83)). Active elbow extension deficiency was recorded in 82 patients (median 25° (5–80)). Elbow flexion strength of the affected side was uniformly impaired (median ratio 43% (0–79)). Median active extension of the wrist was 55° (-70–90). The median ratio of grip strength for the affected side vs. the unaffected side was 68% (0–121). Patients with total injury had poorer ROM and strength than those with C5–6 injury. Incongruity of the radiohumeral joint and avulsion were associated with poor strength values.

**Interpretation:**

ROM and strength of affected upper limbs of patients with surgically treated brachial plexus birth palsy were reduced. Patients with avulsion injuries and/or consequent joint deformities fared worst.

Most brachial plexus birth palsy (BPBP) patients (66–92%) recover spontaneously (Michelow et al. 1994, Noetzel et al. 2001, Hoeksma et al. 2004, Pöyhiä et al. 2010). Indications for brachial plexus surgery vary (Kay 1998, Rust 2000, O'Brian et al. 2006). However, severe total injury or upper-middle plexus injury with no signs of spontaneous recovery within 3–6 months is widely accepted as an indication for early operative treatment (Gilbert et al. 1988, Clarke and Curtis 1995, Strömbeck et al. 2000, Smith et al. 2004).

The severity of neural involvement in BPBP varies from transient neurapraxia to avulsion-type root injuries. Upper plexus (C5-6) injury affects shoulder and elbow function. Furthermore, wrist function is affected to varying degrees in more extensive injuries that involve the upper and middle plexus (C5-7). In total injuries (C5-T1), finger function is also compromised (Bager 1997, Sheburn et al. 1997).

Muscle weakness and joint contractures of the affected upper limb are common in patients with permanent BPBP (Zancolli 1981, Waters et al. 1998, Hoeksma et al. 2003, Kirjavainen et al. 2007, Strömbeck et al. 2007). Muscle imbalance in BPBP patients can lead to soft tissue contractures and eventually to joint deformities (Pollock and Reed 1989, Waters et al. 1998, Nath et al. 2007). There is a negative correlation between degree of osseous deformity of the glenohumeral joint and shoulder range of motion (ROM) (Hoeksma et al. 2003, Kozin 2004).

In this population-based, cross–sectional, long-term follow-up study, we assessed ROM and isometric maximal muscle strength of the upper limbs of surgically treated BPBP patients.

## Patients and methods

We performed a population-based, cross-sectional follow-up study on data from 107 patients treated for BPBP; these data were obtained from the hospital discharge register of the National Research and Development Center for Welfare and Health (STAKES) for 1971 through 1998. 1,706 patients had received treatment for BPBP, of which 124 had had brachial plexus surgery over the 27 year period. Of these patients, 3 were excluded from the study: 2 due to hemiparesis that resulted from cerebral palsy and 1 because of mental retardation. The remaining 121 patients were invited to participate in this study and 107 (64 females) agreed. All physical examinations were performed in the Hospital for Children and Adolescents, Helsinki University Central Hospital, between September 2002 and October 2003. This study was part of a larger investigation carried out at Helsinki University Central Hospital.

### Patients

The median ages of the patients at the time of brachial plexus surgery were 2.9 (0.4–11.0) months, 2.2 (0.9–6.8) months, and 2.4 (0.8–4.9) months for the C5-6 injury group, the C5-7 injury group, and the total injury group (see below). Direct neuroraphy was the most common procedure (n = 65). Lower plexus reconstruction was performed on one fifth of the patients who had total palsy. For the remaining patients with total palsy, either upper or middle plexus reconstruction was performed. Physical examinations were carried out at a median of 12 (5–32) years after the primary surgery. The right upper limb was affected in 56 of the patients and the left upper limb in 51. 3 patients had temporary contralateral BPBP. The extent of the injury was estimated by physical examination, electromyography (EMG), cervical myelography, and intraoperative findings. Of the 107 patients, 51 had a C5-6 palsy, 31 had a C5-7 palsy, and 25 had a total brachial plexus palsy. None of the patients had isolated lower nerve root injury. Root avulsion was diagnosed in 34 patients by intraoperative findings or by myelography. The numbers of patients who had avulsion involvement were: 17 for 1 root, 11 for 2 roots, 3 for 3 roots, 1 for 4 roots, and 1 for 5 roots. No other illness causing contracture of the joints or muscle atrophy of the upper limb was noted in these patients. During the follow-up period, secondary reconstructive procedures were performed on the shoulders or forearms, or hands of 59 patients. 29 patients underwent more than one secondary operation (Table 1).

**Table 1.** Types of secondary procedures performed during the follow-up period

Extent of injury	Median age in years at operation (range)	Median number of procedures (range)	Soft-tissue shoulder procedure	Humeral osteotomy	Soft-tissue forearm procedure	Bone forearm procedure	Hand procedure
C5–6 (n=51)	7 (1–13)	1 (1–3)	15	6	4	0	0
C5–7 (n=31)	5 (2–11)	2 (1–4)	16	3	5	2	5
Total (n=25)	6 (5–11)	2 (1–3)	6	3	11	3	5
Sum			37	12	20	5	10

### Physical examination

Passive and active ROM measurements of the affected and the unaffected upper limbs were taken by two unblinded physiotherapists. They used a standard goniometer, which was marked in 1-degree divisions. External rotation in adduction, abduction, flexion, and extension of the shoulder, elbow flexion, elbow extension, forearm pronation and supination, extension, flexion, and ulnar and radial deviation of the wrist were measured. Radial abduction of the thumb was also recorded.

Maximum isometric muscle strength was measured using a Jamar dynamometer (Asimow Engineering Co., Los Angeles, CA) and also a Good Strength Metitur adjustable dynamometer chair (Metitur Oy, Jyväskylä, Finland) (Figure). The validity and reliability of these two devices have been reported (Mathiowetz et al. 1984, Hovi et al. 1993, Era et al.1994, Bellace et al. 2000, Curb et al. 2006). Shoulder (external and internal rotation strength in adduction), elbow (flexion and extension strength), and grip strength measurements were obtained from patients in a sitting position on a custom-made dynamometer chair. Grip strength was measured using the Jamar dynamometer and the Good Strength dynamometer chair for comparison. 3 measurements each for the affected and the unaffected sides were taken and the best attempt was taken as the result. Ratios of the strength of the unaffected hand to that of the affected hand were calculated and values of less than 89% were judged to be subnormal in further analysis (Petersen et al. 1989). The strength ratio was also evaluated in relation to the patient's age.

**Figure:**
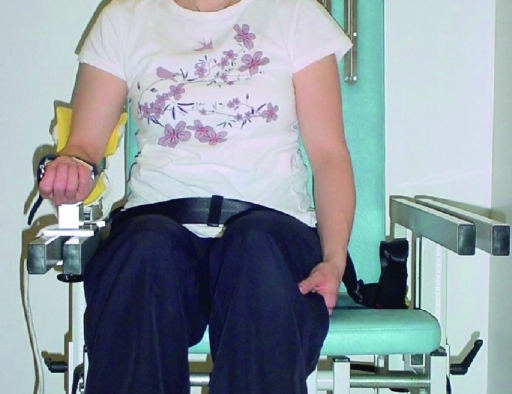
The Metitur Good Strength device for measurement of elbow flexion strength.

Radiographs of the shoulder (anteroposterior AP and axillary) and elbow (AP and lateral) were taken in 95 patients. 2 patients were pregnant and 10 patients declined. Congruity of the glenohumeral joint was classified according to an arbitrary scale: (1) congruent, (2) posteriorly subluxated, (3) posteriorly dislocated, (4) anteriorly subluxated, and (5) anteriorly dislocated. Similarly, congruency of the radiohumeral joint was estimated by evaluating the radiographs as follows: (1) congruent, (2) subluxated, and (3) dislocated. Some of these radiographic data have been reported in an earlier study (Kirjavainen et al. 2007).

### Statistics

Values are expressed as median (range). We used Mann-Whitney U-test to examine the differences for continuous variables and chi-squared test for categorical variables. Correlations were analyzed using the Spearman rank correlation test. 2-tailed p-values of less than 0.05 were considered statistically significant. Strength and ROM measurements were correlated with the type of injury (avulsion vs. no avulsion), secondary procedures, and the congruity of glenohumeral and radiohumeral joint by univariate analysis. Odds ratios (ORs) and their 95% confidence intervals (CIs) were used to analyze the presence of good clinical outcome for patient injury groups using multivariate analysis logistic regression models (NCSS statistical software version 6.0; NCSS, Kaysville, UT). Age at the time of the operation, sex, type of injury (avulsion vs. no avulsion), and congruity of the glenohumeral and radiohumeral joints were considered independent covariates in these analyses. The type of surgery (neurolysis, neuroraphy, grafting, or neurotization) was closely associated with the type (avulsion vs. no avulsion) or with the extent of the neural injury. Thus, both of these covariates could not be included in the same logistic regression model.

### Ethics

The Ethics Research Board of Helsinki University Central Hospital approved this study (no. 79/E7/2001). Permission to contact the subjects was received from different hospital districts throughout Finland. A signed informed consent form was obtained from the patients, or from their parents if the patients were minors.

## Results

### The shoulder

#### Range of motion

61 of the 107 patients had limited active external rotation of the affected shoulder (≤ 0°). The extent of the injury did not have an effect on the incidence or absence of external rotation (C5-6: 31/51; C5-7: 12/31; and C5-T1: 18/25). Only those patients with C5-6 injury had a median active abduction ROM value of greater than 90° (median 125°). Moreover, 29 patients had no active extension of the shoulder. The shoulder flexion was the best preserved function. The ratios of passive ROM values between the affected and the unaffected sides were well preserved, apart from the external rotation of the shoulder (41% for C5-6, 56% for C5-7, and 43% for C5-T1). The ROM and ratios of active motion for the extent of injury are given in Table 2.

**Table 2.** Median active range of motion (ROM) of the affected upper limb and the ratio (%) between affected and unaffected limb according to the extent of injury

	C5-6		C5-7		Total	
	ROM	Ratio	ROM	Ratio	ROM	Ratio
Shoulder						
External rotation in adduction	–8	0	7	11	–15	0
Abduction	125	75	68	36	50	28
Extension	31	43	10	17	11	20
Flexion	126	75	82	50	51	44
Elbow and forearm						
Extension deficiency	20	94	25	90	35	44
Flexion	135	94	128	90	128	96
Pronation	80	79	67	58	54	54
Supination	50	71	30	42	0	0
Wrist						
Extension	65	86	44	60	35	47
Flexion	65	100	60	92	44	75
Radial deviation	24	100	0	0	–2	0
Ulnar deviation	40	100	41	78	20	44

#### Strength

The median ratio of 44% (0–147) for internal rotation strength of the shoulder indicated that this was better preserved than that of external rotation, at 28% (0–105). However, only one patient of the C5-7 group and 6 patients (12%) of the C5-6 group reached or surpassed the predefined cut-off ratio of > 89% for normal internal rotation strength measurements. Similarly, normal values for external rotation strength were obtained for 1 of the 25 patients with total injury and 2 of the 51 patients with C5-6 injury. None of the patients in the C5-7 injury group had normal external rotation strength (Table 3).

**Table 3.** Isometric strength (N) measurements using the Metitur Good Strength device. Grip strength was also measured by the Jamar dynamometer (kg). Max. value of 3 attempts

	Median total values			
	Affected side	Unaffected side	Ratio	
			%	Range
Shoulder external rotation	18	70	28	
C5-6	25	66	38	0–100
C5-7	16	72	23	0–81
total	9	74	12	0–105
Shoulder internal rotation	43	103	44	
C5-6	53	99	60	18–147
C5-7	36	118	37	9–96
total	42	118	35	0–62
Elbow flexion	59	151	43	
C5-6	60	133	51	5–90
C5-7	66	157	42	0–88
total	46	158	36	0–62
Elbow extension	55	114	49	
C5-6	69	101	80	5–185
C5-7	44	129	40	4–80
total	34	116	27	0–81
Grip	123	228	68	
C5-6	151	207	88	41–121
C5-7	159	241	64	32–91
total	38	226	21	0–86
Grip (Jamar)	8	21	35	
C5-6	11	20	61	19–111
C5-7	7	23	30	3–80
total	1	20	6	0–33

### The elbow and forearm

#### Range of motion

Lack of full extension of the elbow was the most common restriction. 89 patients had an active extension deficiency (median 25° (5–80)). All except 1 patient with total injury had an active extension deficiency. The incidences of active extension deficiency for the C5-6 and C5-7 injury groups were 78% (40/51) and 81% (25/31). Similarly, a median of 15° (5–55) for passive extension deficiency ROM was found in 82 patients. No differences in passive motion of elbow flexion between the groups were detected, whereas for active motion only a 7% difference between the affected and unaffected sides was found. 12 of the 25 patients with total palsy and only 5 of the 51 with C5-6 injury had lack of active supination (< 0°) (p < 0.001). The incidence for the lack of supination for the C5-7 group was 7/31. The ratio between the affected and unaffected sides for forearm pronation indicated the function was well preserved. The ROM and ratios of active motion for the extent of injury are given in Table 2.

#### Strength

The median ratios of strength between the affected and unaffected sides were 43% (0–90) for elbow flexion and 49% (0–185) for elbow extension (Table 3). None of the patients in the C5-7 injury group or the total injury group exceeded the normal predefined ratio (> 89%) for elbow flexion or for elbow extension strength. 1 patient in the C5-6 group had a normal ratio for elbow flexion and 17 patients had normal ratios for elbow extension strength.

### The wrist and hand

#### Range of motion

Extension and radial deviation of the wrist were impaired most often. 16 patients had no active extension (< 0°) and 46 had no active radial deviation (< 0°). The incidences for the C5-6 group were 3/51 for no active extension and 6/51 for no active radial deviation. The corresponding values for the C5-7 injury group were 5/31 and 21/31. For patients with total palsy, 8/25 had no active extension and 19/25 had no active radial deviation. The incidences for patients with no active radial abduction of the thumb (< 0°) were: 7/25 for the total palsy group, 2/31 for the C5-7 group, and 1/51 for the C5-6 group. The ratios of passive ROM between the affected and unaffected sides were well preserved except for those of radial deviation of the wrist (C5-6: 100%; C5-7: 40%, and C5-T1: 67%). The ROM and ratios of active motion for each injury group are given in Table 2.

#### Strength

Higher grip strength measurements were obtained from the patients by the Good Strength device than by the Jamar dynamometer. The ratio of strength between the affected and the unaffected sides was 68% (0–121) with the Good Strength device and 35% (0–11) with the Jamar dynamometer (p < 0.001). 9 of the 107 patients could not hold and compress the Jamar dynamometer; their results were therefore recorded as zero. However, 5 of the same 9 individuals could produce a positive result using the Good Strength device (Table 3). Age was not related to the strength ratios obtained for any patient group; nor was it associated with any other strength measurement.

### Radiographic findings

Radiographic findings, which were used for further statistical analyses, have been reported in our earlier publication (Kirjavainen et al. 2007), which was based on a slightly larger patient population (n = 112). The congruity of the glenohumeral joint was: 80 congruent, 9 posteriorly subluxated, 3 posteriorly dislocated, and 3 anteriorly subluxated. Anterior dislocation was not noted. Moreover, the radiohumeral joint was congruent in 77 patients, subluxated in 14, and dislocated in 4.

### Predictors of upper extremity function

Univariate analyses showed that there was a negative correlation between avulsion-type injury and grip strength for the Jamar (p = 0.007) and for the Good Strength (p < 0.001) device measurements, but not with any other strength measurements or for ROM. Similarly, the incongruency of the glenohumeral joint was associated with a lack of extension of the shoulder joint (p = 0.02), but not with other ROM or strength measurements. There was an inverse correlation between secondary procedures and elbow flexion strength (p = 0.02) but this did not apply to other strength measurements or to ROM.

The following factors were adjusted in the analyses: age at surgery, sex, type of injury (avulsion vs. no avulsion) and congruency of the glenohumeral joint. After these adjustments were made, the avulsion type of injury was predictive of poor shoulder internal rotation strength (OR = 0.4, CI: 0.14–0.89; p = 0.03), impaired grip strength according to Jamar measurement (OR = 0.3, CI: 0.11–0.76; p = 0.01) and poor elbow flexion strength (OR = 0.3, CI: 0.12–0.79; p = 0.02) (Tables 4 and 5). Similarly, an incongruent radiohumeral joint was associated with a poor outcome for grip strength as measured by the Jamar device (OR = 0.2, CI: 0.035–0.79; p = 0.02) and the Good Strength device (OR = 0.2, CI: 0.073–0.70; p = 0.01), and elbow extension strength (OR = 0.3, CI: 0.093–0.95; p = 0.04) (Table 5).

**Table 4.** Odds ratios (ORs) and their 95% confidence intervals (CIs) for shoulder rotation strength (Good Strength; Metitur). Age at plexus surgery, sex, type of injury, and congruency of glenohumeral joint were considered as independent covariates in logistic regression models. A strength ratio of > 0.89 between the affected and the unaffected side was considered normal

		Shoulder external rotation			Shoulder internal rotation		
		(ratio>0.89)			(ratio >0.89)		
Characteristic	n	OR	95% CI	p-value	OR	95% CI	p-value
Age (months)							
< 3	67	1			1		
> 3	40	1.8	0.66–4.8	0.3	2.2	0.89–5.40	0.09
Sex							
F	65	1			1		
M	42	1.1	0.41–2.9	0.9	1.8	0.79–4.3	0.2
Type of injury							
no avulsion	69	1			1		
avulsion	38	0.4	0.13–1.2	0.09	0.4	0.14–0.89	0.03
Congruency of glenohumeral joint							
yes	80	1			1		
no	15	1.3	0.36–4.7	0.7	2.2	0.69–6.7	0.2

**Table 5.** Odds ratios (ORs) and their 95% confidence intervals (CIs) for isometric elbow and hand strength measured by the Good Strength and Jamar dynamometers. Age at plexus surgery, sex, type of injury, and congruency of radiohumeral joint were considered as independent covariates in the logistic regression models. A strength ratio of > 0.89 between the affected and the unaffected side was considered normal

		Grip strength (Jamar)			Grip strength (GS)			Elbow flexion (GS)			Elbow extension (GS)		
		(> 0.89)			(> 0.89)			(> 0.89)			(> 0.89)		
Characteristic	n	OR	95% CI	p-value	OR	95% CI	p-value	OR	95% CI	p-value	OR	95% CI	p-value
Age (months)													
< 3	67	1			1			1			1		
> 3	40	1.2	0.48–3.0	0.7	0.5	0.56–4.7	0.3	0.6	0.25–1.6	0.3	0.8	0.34–2.02	0.7
Sex													
F	65	1			1			1			1		
M	42	1	0.70–2.4	0.9	1	0.93–8.0	0.07	0.6	0.26–1.5	0.3	1.9	0.81–4.5	0.1
Type of injury													
no avulsion	69	1			1			1			1		
avulsion	38	0.3	0.11–0.76	0.01	0.4	0.16–1.04	0.06	0.3	0.12–0.79	0.01	0.5	0.23–1.3	0.2
Congruency of radiohumeral joint													
yes	89	1			1			1			1		
no	18	0.2	0.035–0.79	0.02	0.2	0.073–0.70	0.01	0.4	0.14–1.7	0.3	0.3	0.093–0.95	0.04

## Discussion

Patients with permanent BPBP have various degrees of functional impairment of their affected upper extremities, due to a limited ROM and a reduction in muscle power (Hardy 1981, Boome and Kaye 1988, Hoeksma et al. 2003). Secondary joint procedures have been reported to improve function in patients with permanent palsy (Pearl et al. 2006, Waters et al. 2006, 2008). Comparing the results obtained from different institutions is difficult, as all the commonly used scales for ROM and strength of the affected upper limb in BPBP are descriptive: Mallet classification, Toronto test score, and active movement scale (Bae et al. 2003).

Our patients were relatively young at the time of plexus surgery, which—along with the small size of the child—possibly facilitated end-to-end-type repair by neuroraphy. Direct neuroraphy was used more often in patients with C5-6 injury than in those with total injury. In contrast, grafting and neurotization were used more often in patients with total injury. Root avulsion had been sustained in almost one-third of the patients in our study.

The strength of our study is the long-term follow-up. On the other hand, the 27-year-long patient inclusion period, which lasted from 1971 to 1998, can be considered a weakness—as indications, timing, and surgical techniques for primary plexus reconstruction and secondary procedures have changed over such a long time. The long-term results of patients with or without secondary operations cannot be compared with each other, due to the cross-sectional study design. Furthermore, caution should be exercised when the results of this nationwide investigation are compared with results from modern tertiary institutions specialized in the treatment of BPBP.

### The shoulder

A strong association between shoulder contracture and osseous deformity was reported by Hoeksma et al. (2003). Half of their patients had a shoulder contracture of > 10°. We found a correlation between the incongruency of the glenohumeral joint and the poor extension of the shoulder. A lack active external rotation was the most common shoulder impairment and was found in more than half of our patients, which supports the findings by Hoeksma et al. (2003). Pagnotta et al. (2004) reported a series of 203 patients who had undergone latissimus dorsi transfer for restoring shoulder abduction and external rotation with 10 to 15-years of follow-up. These authors obtained satisfactory results with active external rotation. They also noted that this improvement was sustained after the procedure for all patient groups—except the C5-6 group, in which they found that a slight deterioration had occurred between 10 and 15 years of follow-up. On the other hand, they found progressive deterioration of active abduction after 6–10 years of surgery. They also reported a mean abduction of 105° for the C5-6 group and 75° for the C5-7 group at 15 years of follow–up, as compared to only 68° for the total injury group at 10 years of follow-up. The median values for the corresponding injury groups in our more heterogeneous population were 125°, 68°, and 50° at 12 years of follow-up.

The results of a 30-year-long follow-up study by Kirkos et al. (2005) also suggest that short-term satisfactory results with soft tissue procedures to restore active abduction and external rotation of the shoulder were not maintained. According to our results, patients with secondary reconstructive procedures generally had poorer ROM values than those patients who had been treated with primary plexus surgery. This finding can probably be partly explained by those patients with plexus surgery alone having sustained a less severe BPBP than patients who subsequently underwent secondary operations.

To our knowledge, there have been no previous reports in the literature on shoulder strength measurements in operatively treated patients with BPBP. In the present study, both external and internal rotation strength were markedly diminished. Patients with an avulsion-type injury had poorer strength in internal rotation than those patients without root avulsions, which seems logical since patients with root avulsions have potentially fewer motor neurons.

### The elbow and forearm

A Swedish study reported that 90% of all patients with permanent BPBP had an elbow extension deficit (Strömbeck et al. 2007). The authors also found that elbow extension deteriorated substantially during the 5-year-long follow-up period, whereas shoulder and forearm rotation remained unchanged. Restriction of rotation of the active forearm was reported by Sibinski et al. (2007) who found that most of their patients had subnormal (< 80° ROM) active pronation and active supination. We found extension deficit in 89% of all our patients and a lack of active supination in 56% of them, which is in accordance with earlier studies. Both elbow flexion and extension strength were markedly reduced in our patients (usually with > 50% reduction). Furthermore, we found that none of the patients with C5-7 or total injury reached the normal ratio for these strength measurements.

### The wrist and hand

Changes in hand and wrist movements have been reported in patients with either C5-7 or total injury (Strömbeck et al. 2007). We found limited wrist and hand ROM mainly in the C5-7 group and the total injury group. However, we also found that 5 patients (10%) with C5-6 palsy had limited hand and wrist ROM. This finding indicates that such patients may have had a more extensive plexus lesion than that in an upper root injury, which reflects the difficulty in reliably determining the extent of the root injury.

Recently, Strömbeck et al. (2007) found that grip strength as measured by the Jamar dynamometer was subnormal (ratio 80%) for all 12 patients with total injury. Their finding is in line with that in our series, where we found that none of the patients with total injury attained or exceeded the normal predefined ratio (> 89%) as measured by either the Jamar dynamometer or the Good Strength device. However, these authors also reported that 22% of patients with C5-6 injury (9/41) had subnormal grip strength. Their finding is similar to the 34% (Jamar) and 14% (Good Strength) that we found. The differences between our study data and theirs might be explained by the more lenient criteria they used for defining normal grip strength, which perhaps underestimated the numbers of individuals with subnormal grip strength. In our study, grip strength measurement was higher using the Good Strength device than that measured by the Jamar dynamometer. This is probably due to the fixed platform of the Good Strength device, which gives support to the arm.

In the present study, ROM and strength of the shoulder and elbow reflect the outcome of BPBP after plexus reconstruction and also secondary surgery, while in every patient's upper nerve root (responsible for shoulder and elbow) neuroma (5–6) was cut/resected and after that reconstructed. Later on perhaps secundary surgery may have performed. However, the results for hand function mainly reflected the natural history of lower root recovery in total injuries, as most patients with total injury had only upper or middle plexus reconstruction. Median strength of the affected upper limb was less than 50% of that of the unaffected upper limb in every measurement except for grip strength, as measured by the Good Strength device. The poorest results were obtained for external rotation strength of the shoulder. Active external rotation ROM of the shoulder was the most adversely affected parameter, with 0° as the median value. As expected, passive movements were generally better maintained than active movements. Avulsion-type injury and incongruent radiohumeral joint were associated with poor results for ROM and strength measurements.

## References

[CIT0001] Bae DS, Waters PM, Zurakowski D (2003). Reliability of three classification systems measuring active motion in brachial plexus birth palsy. J Bone Joint Surg (Am).

[CIT0002] Bager B (1997). Perinatally acquired brachial plexus palsy – a persisting challenge. Acta Peaditr.

[CIT0003] Bellace JV, Healy D, Besser MP, Byron T, Hohman L (2000). Validity of the Dexter evaluation system's Jamar dynamometer attachment for assessment of the hand grip strength in a normal population. J Hand Ther.

[CIT0004] Boome RS, Kaye JC (1988). Obstetric traction injuries of the brachial plexus. Natural history, indication for surgical repair and results. J Bone Joint Surg (Br).

[CIT0005] Clarke HM, Curtis CG (1995). An approach to obstetrical brachial plexus injuries. Hand Clin.

[CIT0006] Curb JD, Ceria-Ulep CD, Rodriguez BL, Grove J, Guralnik J, Willcox BJ, Donlon TA, Masaki KH, Chen R (2006). Performance-based measures of physical function for high-function populations. J Am Geriatr Soc.

[CIT0007] Era P, Rantanen T, Avlund K, Gause-Nilsson I, Heikkinen E, Schroll M, Steen B, Suominen H (1994). Maximal isometric strength and antropometry among 75-year-old men and women in three Nordic localities. Scand J Med Sci Sports.

[CIT0008] Gilbert A, Razaboni R, Amar-Khodja S (1988). Indications and results of brachial plexus surgery in obstetrical palsy. Orthop Clin North Am.

[CIT0009] Hardy AE (1981). Birth injuries of the brachial plexus. Incidence and prognosis. J Bone Joint Surg (Br).

[CIT0010] Hoeksma AF, ter Steeg AM, Dijkstra P, Nelissen RG, Beelen A, de Jong BA (2003). Shoulder contracture and osseous deformity in obstetrical brachial plexus injury. J Bone Joint Surg (Am).

[CIT0011] Hoeksma AF, ter Steeg AM, Nelissen RG, van Ouwerkerk WJ, Lankhorst GJ, de Jong BA (2004). Neurological recovery in obstetrical brachial plexus injuries: an historical cohort study. Dev Med Child Neurol.

[CIT0012] Hovi L, Era P, Rautonen J, Siimes MA (1993). Impaired muscle strength in female adolescents and young adults surviving leukemia in childhood. Cancer.

[CIT0013] Kay SP (1998). Obstetrical brachial palsy. Br J Plast Surg.

[CIT0014] Kirjavainen M, Remes V, Peltonen J, Kinnunen P, Pöyhiä T, Telaranta T, Alanen M, Helenius I, Nietosvaara Y (2007). Long-term results of obstetrical brachial plexus surgery – a population- based clinical and radiological study. J Bone Joint Surg (Am).

[CIT0015] Kirkos JM, Kyrkos MJ, Kapetanos GA, Haritidis JH (2005). Brachial plexus palsy secondary to birth injuries. J Bone Joint Surg (Br).

[CIT0016] Kozin SH (2004). Correlation between external rotation of the glenohumeral joint and deformity after brachial plexus birth palsy. J Pediatr Orthop.

[CIT0017] Mathiowetz V, Weber K, Volland G, Kashman N (1984). Reliability and validity of grip and pinch strength evaluations. J Hand Surg (Am).

[CIT0018] Michelow BJ, Clarke HM, Curtis CG, Zuker RM, Seifu Y, Andrews DF (1994). The natural history of obstetrical brachial plexus palsy. Plast Reconstr Surg.

[CIT0019] Nath RK, Lyons AB, Melcher SE, Paizi M (2007). Surgical correction of the medial rotation contracture in obstetric brachial plexus palsy. J Bone Joint Surg (Br).

[CIT0020] Noetzel MJ, Park TS, Robinson S, Kaufman B (2001). Prospective study of recovery following neonatal brachial plexus injury. J Child Neurol.

[CIT0021] O'Brien DF, Park TS, Noetzel MJ, Weatherly T (2006). Management of birth brachial plexus palsy. Childs Nerv Syst.

[CIT0022] Pagnotta A, Haerle M, Gilbert A (2004). Long-term results on abduction and external rotation of the shoulder after latissimus dorsi transfer for sequelae of obstetric palsy. Clin Orthop.

[CIT0023] Pearl ML, Edgerton BW, Kazimiroff PA, Burchette RJ, Wong K (2006). Arthroscopic release and latissimus dorsi transfer for shoulder internal rotation contractures and glenohumeral deformity secondary to brachial plexus birth palsy. Bone Joint Surg (Am).

[CIT0024] Petersen P, Petrick M, Connor H, Conklin D (1989). Grip strength and hand dominance: challenging the 10% rule. Am J Occup Ther.

[CIT0025] Pollock AN, Reed MH (1989). Shoulder deformities from obstetrical brachial plexus paralysis. Skeletal Radiol.

[CIT0026] Pöyhiä TH, Lamminen AE, Peltonen JI, Kirjavainen MO, Willamo PJ, Nietosvaara YA (2010). Brachial plexus birth injury: US screening for glenohumeral joint instability. Radiology.

[CIT0027] Rust RS (2000). Congenital brachial plexus palsy: Where have we been and where are we now?. Semin Pediatr Neurol.

[CIT0028] Sherburn EW, Kaplan SS, Kaufman BA, Noetzel MJ, Park TS (1997). Outcome of surgically treated birth-related brachial plexus injuries in twenty cases. Pediatr Neurosurg.

[CIT0029] Sibinski M, Sherlock DA, Hems TE, Sharma H (2007). Forearm rotational profile in obstetric brachial plexus injury. J Shoulder Elbow Surg.

[CIT0030] Smith NC, Rowan P, Benson LJ, Ezaki M, Carter PR (2004). Neonatal brachial plexus palsy. Outcome of absent biceps function at three months of age. J Bone Joint Surg (Am).

[CIT0031] Strömbeck C, Krumlinde-Sundholm L, Forssberg H (2000). Functional outcome at 5 years in children with obstetrical brachial plexus palsy with and without microsurgical reconstruction. Dev Med Child Neurol.

[CIT0032] Strömbeck C, Krumlinde-Sundholm L, Remahl S, Sejersen T (2007). Long-term follow-up of children with obstetric brachial plexus palsy I: functional aspects. Dev Med Child Neurol.

[CIT0033] Waters PM, Smith GR, Jaramillo D (1998). Glenohumeral deformity secondary to brachial plexus birth palsy. J Bone Joint Surg (Am).

[CIT0034] Waters PM, Bae DS (2006). The effect of derotational humeral osteotomy on global shoulder function in brachial plexus birth palsy. J Bone Joint Surg (Am).

[CIT0035] Waters PM, Bae DS (2008). The early effects of tendon transfers and open capsulorrhaphy on glenohumeral deformity in brachial plexus birth palsy. J Bone Joint Surg (Am).

[CIT0036] Zancolli EA (1981). Classification and management of the shoulder in birth palsy. Orthop Clin North (Am).

